# Application of Osteorisk to postmenopausal patients with osteoporosis

**DOI:** 10.1590/S1516-31802010000100006

**Published:** 2010-01-07

**Authors:** Marcelo Luis Steiner, César Eduardo Fernandes, Rodolfo Strufaldi, Everaldo Cunha Porto, Luciano de Melo Pompei, Sérgio Peixoto

**Affiliations:** I MD. Assistant lecturer, Department of Gynecology and Obstetrics, Faculdade de Medicina do ABC (FMABC), São Bernardo do Campo, São Paulo, Brazil.; II MD, MSc, PhD. Head of the Endocrine Gynecology and Climacterium Sector, Department of Gynecology and Obstetrics, Faculdade de Medicina do ABC (FMABC), São Bernardo do Campo, São Paulo, Brazil.; III MD. Attending physician, Gynecology and Climacterium Sector, Department of Gynecology and Obstetrics, Faculdade de Medicina do ABC, São Bernardo do Campo, São Paulo, Brazil.; IV MD, MSc. Attending physician, Endocrine Gynecology and Climacterium Sector, Department of Gynecology and Obstetrics, Faculdade de Medicina do ABC (FMABC), São Bernardo do Campo, São Paulo, Brazil.; V MD, MSc, PhD. Titular professor, Department of Gynecology and Obstetrics, Faculdade Medicina do ABC (FMABC), São Bernardo do Campo, São Paulo, Brazil.

**Keywords:** Bone mineral density, Densitometry, Menopause, Osteoporosis, Risk factors, Densidade óssea, Densitometria, Menopausa, Osteoporose, Fatores de risco

## Abstract

**CONTEXT AND OBJECTIVE:**

Identification of women at risk of bone fracture is becoming less dependent on evaluating bone mineral density through placing greater value on clinical risk factors. The aim of this study was to evaluate the sensitivity of the Osteorisk clinical tool for identifying Brazilian postmenopausal women with osteoporosis, compared with bone densitometry.

**DESIGN AND SETTING:**

Cross-sectional observational study at Faculdade de Medicina do ABC.

**METHOD:**

Information on 812 postmenopausal osteoporotic women was retrospectively evaluated from medical records. The women were divided into the age groups 50-59, 60-69, 70-79 and over 80 years. The results from the Osteorisk clinical tool, which uses only age and weight, were compared with bone densitometry T-scores.

**RESULTS:**

There were significant correlations between the results from the Osteorisk clinical tool and from bone densitometry, in relation to the lumbar spine (P = 0.027) and hip (P < 0.001), thus showing a non-arbitrary relationship. The overall sensitivity of Osteorisk for identifying women with “high risk of osteoporosis” was 86.5%, and it was higher for hip osteoporosis alone (97.2%) than for lumbar spine osteoporosis (85.8%). The sensitivity was better among older women.

**CONCLUSION:**

Osteorisk seems to present good sensitivity for identifying postmenopausal women at risk of osteoporosis. It should be used when bone densitometry is not easily available or as a means of selecting individuals for referral for bone densitometry.

## RESUMO

**CONTEXTO E OBJETIVO:**

A identificação de mulheres com risco para fratura está ficando cada vez menos dependente da avaliação da densidade mineral óssea com a valorização dos fatores clínicos de risco. O objetivo deste estudo foi avaliar a sensibilidade da ferramenta clínica Osteorisk em identificar mulheres menopausadas e com osteoporose quando comparada com a densitometria óssea.

**TIPO DE ESTUDO E LOCAL:**

Estudo observacional do tipo transversal realizado na Faculdade de Medicina do ABC.

**MÉTODO:**

Foram avaliadas retrospectivamente informações de 812 mulheres na pós-menopausa e com osteoporose por meio dos prontuários clínicos. Elas foram divididas nos grupos etários 50-59, 60-69, 70-79 e mais de 80 anos e os resultados da ferramenta clínica Osteorisk, que utiliza apenas a idade e o peso, foram comparados com os valores de T-scores da densitometria óssea.

**RESULTADOS:**

Houve correlação entre os resultados da ferramenta clínica Osteorisk e da densitometria óssea, em relação à coluna lombar (P = 0,027) e quadril (P < 0,001), mostrando assim uma relação não-arbitrária. A sensibilidade geral do Osteorisk para identificar mulheres com “alto risco de osteoporose” foi 86,5% e superior para identificar osteoporose de quadril (97,2%) do que para a coluna lombar (85,8%). A sensibilidade foi maior nas mulheres idosas.

**CONCLUSÃO:**

Osteorisk parece apresentar boa sensibilidade para identificar mulheres na pós-menopausa em risco de osteoporose. Deveria ser usado quando a densitometria óssea não é facilmente disponível ou como um meio de seleção de indivíduos para realizar a densitometria óssea.

## INTRODUCTION

The prevalence of osteoporosis, along with the fractures consequent to it, is increasing year by year. It has been estimated that, with the aging of the population in Latin America, the number of hip fractures among men and women aged 50 to 60 years will be 400% greater in the year 2050, in comparison with 1950, and almost 700% greater among individuals over the age of 65 years.^1^

There is an increasing need for diagnosis and therapeutic intervention among climacteric women who present a risk of fractures due to bone fragility, in order to save them from the morbidity and mortality caused by this event. Over the last two decades, diagnoses and prognoses of osteoporosis have been made based on measurements of bone mineral density (BMD), particularly because this is the single most important factor for preventing fractures.^2^ There are various technologies available for evaluating BMD.^3,4^ However, the technique that is considered to be the gold standard is dual-energy x-ray absorptiometry (DXA).^5-9^

DXA is the complementary examination most used in clinical trials to evaluate therapy for osteoporosis because it is fast, effective and safe. Epidemiological studies have found an excellent correlation between the risk of fractures and BMD assessed by DXA.^5,10,11^ Thus, for good fracture prevention, it would be desirable for all climacteric women to undergo DXA and then to those with a diagnosis of osteoporosis receive treatment. However, applying DXA indiscriminately to the whole population of climacteric women with the purpose of diminishing the fracture rate would have a high cost and questionable effectiveness. Moreover, it would be limited to geographical areas where the DXA apparatus is available.^2,3,5^

High investment could lead to a real decrease in the number of fractures and thus compensate for the expenditure that would be destined for treating them. Nevertheless, despite observing more and more that measurements using DXA enable precise diagnosis of BMD and are useful for monitoring treatments, DXA is inefficient for identifying which women are going to present fractures, when considered alone.^12-14^

In the current literature, it is increasingly evident that DXA is used as an additional risk factor and not as the main criterion for establishing the approach in osteoporosis cases. This is clear in the Fracture Assessment Tool (FRAX), a clinical tool devised by the World Health Organization (WHO) that has the capacity to identify the likelihood of fractures over the next 10 years, even without measuring BMD, based on clinical risk factors.^15^ Thus, the questions that arise are when to request DXA and how to make it more sensitive with regard to identifying women at greater risk of osteoporosis.

There is a growing trend towards using risk factors for osteoporosis and fractures as a screening method for measuring BMD. The aim is to improve the sensitivity of identifying women at increased risk of osteoporosis.^16-21^

Using this reasoning, in 2005, Sen et al. presented a clinical tool to aid in indicating the need for performing DXA among women in Latin America, called Osteorisk.^9^ The proposal consisted of a simple mathematical formula using weight and age to classify postmenopausal women as presenting low, medium or high risk of osteoporosis. The women in the high and medium-risk groups are considered more likely to present bone fragility and therefore they should be referred for DXA.

## OBJECTIVE

Perceiving the value of this clinical tool, our objective in this study was to evaluate its sensitivity for identifying Brazilian postmenopausal women with a densitometric diagnosis of osteoporosis.

## METHOD

### Population

The patients were selected from the databases of the Institute of Women's Health and Wellbeing (Instituto de Saúde e Bem Estar da Mulher, ISBEM), located in São Paulo, and the Center for Integrated Women's Healthcare (Centro de Atenção Integral a Saúde da Mulher, CAISM), located in São Bernardo do Campo, a city within the metropolitan area of São Paulo. The study protocol covered both centers and had previously been submitted to the Research Ethics Committee of the Faculdade de Medicina do ABC (FMABC), gaining unrestricted approval.

Between September 2004 and October 2006, 1,253 postmenopausal women were evaluated and, of these, 812 presented the criteria for participation in this study. For inclusion, the women had to be over 50 years old and had to have reached the menopause naturally or surgically (bilateral oophorectomy with or without hysterectomy), with or without climacteric symptoms. They had to present a diagnosis of osteoporosis from densitometric evaluation, in accordance with the World Health Organization (WHO) criteria.^22,23^ It was required that, over the preceding six months, they had not made use of any of the following medications: estrogens, progestogens, androgens, calcitonin, bisphosphonates, aromatase inhibitors, calcium, heparin, selective estrogen receptor modulators (SERMs), parathyroid hormone (PTH), anticonvulsants (phenobarbital, primidone, phenytoin, carbamazepine or topiramate), lithium or corticosteroids equivalent to 10 mg/day of prednisone for periods longer than 10 days. In addition, patients who were drug or alcohol abusers or who had used abusive doses of antacids over the same period were excluded.

The women were characterized as postmenopausal if they had not menstruated for at least 12 months or if they presented follicle-stimulating hormone (FSH) levels greater than 30.0 UI/l.^24^

Patients presenting any of the following were excluded: complains of intense bone pain, bone implants, antecedents of non-traumatic fractures, bone metabolic dysfunction (hypercalcemia, hypocalcemia, *osteogenesis imperfecta*, chronic gastrointestinal disease, Paget's disease or hyperparathyroidism), antecedents of bone metastases, thyroid diseases, presence of nephropathy and lack of densitometric diagnosis of osteoporosis in the lumbar spine or femoral neck.

The database for this cross-sectional study was built up by reviewing the medical files of patients who attended the climacterium and osteoporosis outpatient clinics of ISBEM and CAISM. All the medical files between September 2004 and October 2006 were surveyed, and 1253 files containing the information needed for carrying out this study were identified. After detailed analysis of these files with regard to the parameters defined for inclusion in the study, it was concluded that 812 presented the criteria for participation.

### Bone mineral density evaluation

From analysis of the result reports from bone densitometry examinations, patients with osteoporosis in the vertebral column or femoral neck, or both, were selected. These patients’ medical files were then analyzed using the densitometric criterion, with the aim of verifying whether they were also within the inclusion criteria determined for this study.

All the patients’ ages, weights in kilograms and heights in meters were recorded at the time they underwent the bone densitometry examination.

The BMD measurements of the lumbar spine and femoral neck among the patients at both centers were performed using the Lunar DPX IQ or Lunar DPX alpha apparatus, which use the same software for data processing and analysis. The coefficient of variation of both types of apparatus was 1.1%. The examinations were performed by trained technicians and all the results were presented as computer-generated reports.

The patients were considered osteoporotic when the bone densitometry examination showed that the BMD in the lumbar spine or femoral neck was lower than or equal to −2.5 standard deviations below the BMD of young adults.

We chose to measure the BMD of the lumbar spine in the segment comprising the lumbar vertebrae one to four (L1 to L4) and preferentially the BMD of the right-side femoral neck. In the event of any technical or physical difficulty presented by the patient, the left side was used.

### Anthropometry

Weights and heights were measured using an anthropometric balance, with the patients in the orthostatic position. The body mass index (BMI) was calculated by dividing the weight in kilograms by the square of the height in meters.^25^

### Osteorisk

Osteorisk was developed by means of statistical multivariate regression calculations to predict the BMD. The potential risk factors used to develop the index were: age, weight, use of postmenopausal hormonal therapy, history of use of thyroid hormone, history of fractures after the age of 45 years, ethnicity, country of residence and BMI.

The model with all the variables presented sensitivity of 91% and specificity of 46% for identifying women with osteoporosis. After analyzing all the combinations of variables, it was concluded that the simplest model that would satisfy the preestablished performance criterion (sensitivity > 85% and specificity > 45%) contained only age and body weight. Based on this model, Osteorisk was calculated as 0.2 X [(weight in kg) – (age in years)]. Individuals were considered to be at low risk when the calculation gave a value greater than 1, at medium risk when it was between −2 and 1 and at high risk when it was lower than −2.

To evaluate the sensitivity of Osteorisk in relation to the T-score, we established that:

- Patients without risk would be the ones presenting low risk in Osteorisk.
- Patients with risk would be all others (medium or high risk in Osteorisk).

### Statistical analysis

The sample size was determined by a convenience sample. With a sample size of 812 and a power of 80%, the chance that Osteorisk would detect a high risk of osteoporosis was more than 86.5%, comparable with the literature.

The data were summarized as means ± standard deviation for quantitative variables and as absolute frequencies (n) and relative frequencies (%) for the qualitative variable.

The presence of an association between the Osteorisk classification and the T-scores for the lumbar spine (L1-L4) and the femoral neck was evaluated by means of the generalization of Fisher's exact test.

For all statistical analyses, a significance level of 5% was adopted (α = 0.05).

## RESULTS

The descriptive analysis on some of the demographic and clinical data, such as age, age group, ethnicity, weight, height, BMI and T-score for the lumbar spine and femoral neck can be seen in Table 1.

**Table 1.** Patients’ clinical characteristics (n = 812)

Age group (years)	n (%)
50-59 years	61 (7.5)
60-69 years	387 (47.7)
70-79 years	306 (37.7)
≥ 80 years	58 (7.1)
Mean ± SD	68.82 ± 6.96
**Ethnicity**	**n (%)**
Caucasian	631 (77.7)
African	169 (20.8)
Asian	12 (1.5)
**Height (meters)**	
Mean ± SD	1.50 ± 0.057
**Weight (kg)**	
Mean ± SD	59.04 ± 9.48
**Body mass index (BMI) in kg/m** ^2^	
Mean ± SD	26.26 ± 3.9

SD = standard deviation.

The mean age of the women included in the study was 68.82 ± 7.0 years, and most of them were over 60 years. As shown in Table 1, the age group from 60 to 69 years accounted for 47.7% of the subjects, while only 7.1% were aged between 50 and 59 years. Caucasian ethnicity was the most frequent type, accounting for 77.7%.

The weights of the women in our sample ranged from 34 to 93 kg, with a mean of 59.04 ± 9.5 kg, and ranged in height from 1.34 to 1.69 m, with a mean of 1.50 ± 0.05 m. The mean BMI was 26.26 ± 3.9 kg/m^2^.

Osteorisk identified that 43.7% of the women were at high risk, 42.7% were at medium risk and only 13.6% were at low risk of osteoporosis. Table 2 presents the Osteorisk distribution in relation to the age groups and shows that, in the high-risk group, 15%, 25.5%, 63% and 97% of the patients were in the age groups of 50 to 59, 60 to 69, 70 to 79 and over 80 years, respectively.

**Table 2.** Distribution of the patients, categorized according to the risk strata of Osteorisk, in relation to the different age groups (n = 812)

	Osteorisk			
	High	Medium	Low	Total
Age group	n = 355	n = 347	n = 110	**n = 812**
50-59 years	9 (15%)	19 (31%)	33 (54%)	**61 (100%)**
60-69 years	98 (25.5%)	219 (56.5%)	70 (18%)	**387 (100%)**
70-79 years	192 (63%)	108 (35%)	6 (2%)	**306 (100%)**
≥ 80 years	56 (97%)	1 (1.5%)	1 (1.5%)	**58 (100%)**

Generalization of Fisher's exact test: P < 0.001.

Tables 3 and 4 present the distribution of the patients in the Osteorisk classification, in relation to the BMD of the lumbar spine and femoral neck respectively. In relation to the lumbar spine, it was observed that 14.3% of the patients with osteoporosis according to their BMD were classified as low risk by Osteorisk*.* The association between the Osteorisk classification and the T-score for the lumbar spine, shown in Table 4, was statistically significant (P = 0.027). The comparison with the T-score for the femoral neck, described in Table 4, demonstrated a statistically significant association (P < 0.001).

**Table 3.** Distribution of the patients, categorized according to the risk strata of Osteorisk, in relation to the different strata of densitometric results for the lumbar spine (L1-L4), expressed as T-scores (n = 812)

	Bone densitometry of the lumbar spine (L1-L4)			
	Normal T-score > −1 SD	Osteopenia T-score: ≤ −1 and ≥ −2.5 SD	Osteoporosis T-score ≤ −2.5 SD	Total
Osteorisk	n = 7	n = 49	n = 756	**n = 812**
High	2 (0.28%)	28 (57.2%)	325 (42.9%)	**355 (43.7%)**
Medium	3 (0.43%)	20 (40.8%)	324 (42.8%)	**347 (42.7%)**
Low	2 (0.28%)	1 (2.0%)	107 (14.3%)	**110 (13.5%)**

Generalization of Fisher's exact test: P = 0.027. SD = standard deviation.

**Table 4.** Distribution of the patients, categorized according to the risk strata of Osteorisk, in relation to the different strata of densitometric results for the femoral neck, expressed as T-scores (n = 812)

	Bone densitometry of the femoral neck			
	Normal T-score > −1 SD	Osteopenia T-score: ≤ −1 and ≥ −2.5 SD	Osteoporosis T-score ≤ −2.5 SD	Total
Osteorisk	n = 100	n = 466	n = 246	**n = 812**
High	12 (12.0%)	175 (37.6%)	168 (68.3%)	**355 (43.7%)**
Medium	48 (48.0%)	228 (48.9%)	71 (28.9%)	**347 (42.7%)**
Low	40 (40.0%)	63 (13.5%)	7 (2.8%)	**110 (13.5%)**

Generalization of Fisher's exact test: P < 0.001. SD = standard deviation.

Table 5 shows the distribution of the Osteorisk results in relation to the T-score information from the lumbar spine and femoral neck. From this, it was found that the general sensitivity of Osteorisk was 86.5%. The false negative rate for the lumbar spine was 14.2% and for the femoral neck, it was 2.8%.

**Table 5.** Evaluation of the sensitivity of Osteorisk in relation to the densitometric diagnosis of osteoporosis in the lumbar spine (L1-L4), femoral neck and L1-L4 spine or femoral neck

	Osteoporosis		
Osteorisk	L1-L4 spine	Femoral neck	L1-L4 spine or femoral neck
With risk	649 (85.8%)	239 (97.2%)	702 (86.5%)
Without risk	107 (14.2%)	7 (2.8%)	110 (13.5%)
**Total**	**756 (100.0%)**	**246 (100.0%)**	**812 (100.0%)**

## DISCUSSION

When the Osteorisk clinical tool was compared with DXA in this study, it presented sensitivity of 86.5% for differentiating women who were at greater risk of osteoporosis, independent of the bone site evaluated. This capacity comes close to the sensitivity found in the original study introducing Osteorisk (92%).^9^

It is worth emphasizing that in the original study, the clinical tool was devised by comparing risk factors only with the T-score for the femoral neck, while the lumbar spine was disregarded. In the present study, 68.3% of the women with osteoporosis in the femoral neck were classified as high risk by Osteorisk, corresponding to sensitivity of 97.2% when only this bone site was evaluated.

There is unanimity in the literature in affirming that fractures of the femoral neck have a worse prognosis, since they cause greater morbidity and mortality.^26^ Thus, the small number of false negatives for the femoral neck gives support to the idea that Osteorisk is clinically useful and effective in daily practice.

On the other hand, when we compared the Osteorisk results with the T-score for the lumbar spine, the number of false positives reached 14.2%. This was why the general sensitivity of this tool was no greater than 86.5%. One of the likely explanations for this may have been precisely the fact that this clinical tool was devised by comparing risk factors only with the T-score for the femoral neck. If perhaps it had been devised using other bone sites, other clinical factors might have been shown to be significant and might have entered the formula. This observation is important and makes us speculate whether a new tool originating from comparisons of risk factors relating to more than one bone site might have greater accuracy in relation to what was demonstrated with Osteorisk.

In the population of the present study, only 8% were less than 60 years old, which causes difficulty in undertaking a definitive analysis in relation to age. However, analysis of the use of this tool in relation to the age groups demonstrated that it had greater capacity to discriminate the risk of low bone mass and osteoporosis, the older the population was. The percentages of high and medium risk identified were 46%, 82%, 98% and 98.5% in the age groups of 50 to 59, 60 to 69, 70 to 79 and over 80 years, respectively. Since the ten-year risk of fractures is directly proportional to age,^2^ there is a lower possibility for patients younger than 60 years to present osteoporotic fractures, which diminished the practical use of this tool in that it did not identify younger women with osteoporosis.

The number of false negatives from this study reached 13.5%, and this needs to be analyzed. It is certainly a point of criticism of this tool. It needs to be asked what the implications would be, if this number of women with the disease failed to be referred for BMD evaluation. Likewise, the risk that these patients might present a fracture and, through this, suffer an impact on their quality of life needs to be assessed. Furthermore, the cost to the state of supporting these women, who would usually become economically inactive, has to be considered.

In a review on studies in which the Osteoporosis Self-Assessment Tool (OST), a variation of Osteorisk for European populations, was applied, Rud et al.^27^ came to the conclusion that there were uncertainties in using this clinical tool. They considered that its capacity for discriminating low BMD in the lumbar spine and in combined regions had little clinical usefulness because of the large number of false positives. On the other hand, in relation to the femoral neck, they considered it to have better capacity, such that the use of this site was of more interest for clinical practice. It is worth emphasizing that, in their review, they did not believe that any of the studies assessed presented the recommendable level of technical quality.

There is no unanimity regarding the Osteorisk tool in the literature. A previous study by Steiner et al.,^28^ comparing Osteorisk with bone density measurements obtained from heel ultrasonometry among 461 women, found sensitivity of only 64%, i.e. relatively low in comparison with the 92% of the original study on Osteorisk. This difference was mainly because the values recommended by WHO for diagnosing osteoporosis via DXA were used for heel ultrasonometry. There are studies proposing that, to have a good association between the two examinations, the ultrasonometry scores should have a different basis.^29^

Another observation is that in devising the Osteorisk tool, its authors excluded the population of black skin color.^9^ In the present study, 21% of the women were black. The impact that this might have caused on the results is difficult to assess and open to criticism. There is no doubt that this is very important, since the value of any clinical tool that excludes an ethnic group, particularly in a country with racial miscegenation like Brazil, will be questioned. Nevertheless, the sensitivity in the present study was close to that of the original study, which makes us believe that even though the black population was included in the original description, the original study has not lost its value and it can be applied to miscegenated populations like the Brazilian population.

Finally, considering that the DXA examination is not widely available within the Brazilian public health system (Sistema Único de Saúde, SUS), or in the health systems of other developing countries, and that performing BMD measurements on all women who have passed the menopause would not be feasible or economically rational, the Osteorisk tool may be a viable and intelligent alternative. According to Silva,^30^ around 480,000 women reach the menopause in Brazil every year. SUS pays 54 million reais per annum (equivalent to 21 million euros) for DXA examinations to be performed. For the examination alone, without including the cost of buying the equipment and the expenditure on technicians, physicians and paramedics, the amount invested reaches 26 million reais per annum (almost 10 million euros). Thus, having a tool with the capacity to identify women who are at risk of osteoporosis, with good sensitivity, would help clinicians to make correct referrals for DXA, thereby avoiding referrals for patients who are considered to present low risk, and saving on unnecessary examinations.

The FRAX tool, devised by WHO, was considered to represent an enormous advance in attending osteoporosis cases, yet it can be criticized. It requires a series of clinical information that is sometimes complicated to quantify, such as the use of corticoids or the amount of alcohol consumed.^15^ Furthermore, it depends on having a computer in the outpatient clinic to make the evaluation. If no computer is available, a large number of tables varying according to the patient's age and ethnicity need to be consulted.

Osteorisk can certainly be criticized and it presents limitations. Therefore, it can be contested. Nevertheless, there is no method for differentiating women who are at risk of osteoporosis that is firmly established and free of criticism. This, together with the simplicity of Osteorisk, means that there may be interest in this method for attending patients with osteoporosis, in order to select patients better for DXA and diminish the costs.

The next step beyond the present study is to include a healthy menopausal population in the Osteorisk analysis. Through this, not only the sensitivity but also the accuracy of this clinical tool could be evaluated.

## CONCLUSION

Because of the good sensitivity of Osteorisk, particularly for bone sites with greater morbidity, its excellent capacity for stratifying the risk among the elderly population, its possibility of application to black populations, its simplicity of implementation, its low cost and the savings that it provides, it meets the requirements to be an excellent screening method for identifying women who are at higher risk of osteoporosis.

## References

[1] Cooper C, Campion G, Melton LJ (1992). Hip fractures in the elderly: a world-wide projection. Osteoporos Int.

[2] Kanis JA, Borgstrom F, De Laet C (2005). Assessment of fracture risk. Osteoporos Int.

[3] Nelson HD, Helfand M, Woolf SH, Allan JD (2002). Screening for postmenopausal osteoporosis: a review of the evidence for the U.S. Preventive Services Task Force. Ann Intern Med.

[4] Cummings SR, Bates D, Black DM (2002). Clinical use of bone densitometry: scientific review. JAMA.

[5] Kanis JA (2002). Diagnosis of osteoporosis and assessment of fracture risk. Lancet.

[6] Black DM, Cummings SR, Genant HK, Nevitt MC, Palermo L, Browner W (1992). Axial and appendicular bone density predict fractures in older women. J Bone Miner Res.

[7] Miller PD, Zapalowski C, Kulak CA, Bilezikian JP (1999). Bone densitometry: the best way to detect osteoporosis and to monitor therapy. J Clin Endocrinol Metab.

[8] Siris ES, Miller PD, Barrett-Connor E (2001). Identification and fracture outcomes of undiagnosed low bone mineral density in postmenopausal women: results from the National Osteoporosis Risk Assessment. JAMA.

[9] Sen SS, Rives VP, Messina OD (2005). A risk assessment tool (OsteoRisk) for identifying Latin American women with osteoporosis. J Gen Intern Med.

[10] Sturtridge W, Lentle B, Hanley DA (1996). Prevention and management of osteoporosis: consensus statements from the Scientific Advisory Board of the Osteoporosis Society of Canada. 2. The use of bone density measurement in the diagnosis and management of osteoporosis. CMAJ.

[11] Lewiecki EM, Borges JLC (2006). Densitometria óssea na prática clínica [Bone density testing in clinical practice]. Arq Bras Endocrinol Metabol.

[12] Kanis JA, Johnell O, Oden A, De Laet C, Jonsson B, Dawson A (2002). Ten-year risk of osteoporotic fracture and the effect of risk factors on screening strategies. Bone.

[13] Burger H, De Laet CE, Weel AE, Hofman A, Pols HA (1999). Added value of bone mineral density in hip fracture risk scores. Bone.

[14] Black DM, Steinbuch M, Palermo L (2001). An assessment tool for predicting fracture risk in postmenopausal women. Osteoporos Int.

[15] Kanis JA FRAX WHO Fracture Risk Assessment Tool. Paper charts.

[16] Johansson H, Oden A, Johnell O (2004). Optimization of BMD measurements to identify high risk groups for treatment--a test analysis. J Bone Miner Res.

[17] Cadarette SM, Jaglal SB, Kreiger N, McIsaac WJ, Darlington GA, Tu JV (2000). Development and validation of the Osteoporosis Risk Assessment Instrument to facilitate selection of women for bone densitometry. CMAJ.

[18] Lydick E, Cook K, Turpin J, Melton M, Stine R, Byrnes C (1998). Development and validation of a simple questionnaire to facilitate identification of women likely to have low bone density. Am J Manag Care.

[19] Koh LK, Sedrine WB, Torralba TP (2001). A simple tool to identify asian women at increased risk of osteoporosis. Osteoporos Int.

[20] Sedrine WB, Chevallier T, Zegels B (2002). Development and assessment of the Osteoporosis Index of Risk (OSIRIS) to facilitate selection of women for bone densitometry. Gynecol Endocrinol.

[21] Geusens P, Hochberg MC, van der Voort DJ (2002). Performance of risk indices for identifying low bone density in postmenopausal women. Mayo Clin Proc.

[22] Leib ES, Lewiecki EM, Binkley N, Hamdy RC (2004). International Society for Clinical Densitometry.. Official positions of the International Society for Clinical Densitometry. J Clin Densitom.

[23] (1994). Assessment of fracture risk and its application to screening for postmenopausal osteoporosis. Report of a WHO Study Group. World Health Organ Tech Rep Ser.

[24] Speroff L, Fritz MA, Speroff L, Fritz MA (2004). Menopause and the perimenopausal transition. Clinical gynecologic endocrinology and infertility.

[25] (1995). Physical status: the use and interpretation of anthropometry. Report of a WHO Expert Committee. World Health Organ Tech Rep Ser.

[26] Riggs BL, Melton LJ (1995). The worldwide problem of osteoporosis: insights afforded by epidemiology. Bone.

[27] Rud B, Hilden J, Hyldstrup L, Hróbjartsson A (2007). Performance of the Osteoporosis Self-Assessment Tool in ruling out low bone mineral density in postmenopausal women: a systematic review. Osteoporos Int.

[28] Steiner ML, Fernandes CE, Strufaldi R (2008). Accuracy study on “Osteorisk”: a new osteoporosis screening clinical tool for women over 50 years old. Sao Paulo Med J.

[29] Vu TT, Nguyen CK, Nguyen TL (2005). Determining the prevalence of osteoporosis and related factors using quantitative ultrasound in Vietnamese adult women. Am J Epidemiol.

[30] Silva LK (2003). Avaliação tecnológica em saúde: densitometria óssea e terapêuticas alternativas na osteoporose pós-menopausa [Technology assessment in health care: bone densitometry and alternatives therapeutical in post-menopausal osteoporosis]. Cad Saúde Pública = Rep Public Health.

